# Educational Applications for Blind and Partially Sighted Pupils Based on Speech Technologies for Serbian

**DOI:** 10.1155/2015/839252

**Published:** 2015-06-01

**Authors:** Branko Lučić, Stevan Ostrogonac, Nataša Vujnović Sedlar, Milan Sečujski

**Affiliations:** ^1^Faculty of Technical Sciences, University of Novi Sad, Trg Dositeja Obradovića 6, 21000 Novi Sad, Serbia; ^2^AlfaNum Ltd., Trg Dositeja Obradovića 6, 21000 Novi Sad, Serbia

## Abstract

The inclusion of persons with disabilities has always represented an important issue. Advancements within the field of computer science have enabled the development of different types of aids, which have significantly improved the quality of life of the disabled. However, for some disabilities, such as visual impairment, the purpose of these aids is to establish an alternative communication channel and thus overcome the user's disability. Speech technologies play the crucial role in this process. This paper presents the ongoing efforts to create a set of educational applications based on speech technologies for Serbian for the early stages of education of blind and partially sighted children. Two educational applications dealing with memory exercises and comprehension of geometrical shapes are presented, along with the initial tests results obtained from research including visually impaired pupils.

## 1. Introduction

Computer games have proved to be a valuable tool for certain educational purposes, especially when bearing in mind their ubiquity in the lives of children and the young [1]. If the ergonomic characteristics of the target population are taken into account during the development of computer games, as well as their specific pedagogical and psychological needs, then a game becomes an instrument for the education and therapy of people with disabilities. As is the case with all computer games, it is necessary to achieve the required level of usability, playability, and effectiveness [2, 3]. Usability is directed towards functionality within the game system, while playability is directed towards the functionality of the system as a whole, and for that reason designers of games aimed at the disabled claim playability to be more important [4, 5]. With the disabled, playability mainly depends on to what extent the game is adapted to the user's ergonomic characteristics. With educational games, high effectiveness is the ultimate goal, because it represents the measure of a game's ability to enhance knowledge acquisition and ability development in every individual [6].

An appropriate level of playability of computer games for blind children has been achieved by alternative representation of a graphical user interface (GUI). Namely, computer games for the mentioned population can be roughly divided, regarding the primary sense that they engage, into audio [7–10] and tactile computer games [11–13], as well as games designed as a combination of the two basic models [14, 15]. Each of the two basic solutions has its own restrictions, and it is not possible to reach the desired level of playability in different computer games while they remain solely audio or solely tactile [16, 17]. For example, games such as action games and adventure games usually require active usage of the eyesight of the player, and as a result, games of this type are scarcely available for the visually impaired [18]. Among computer games aimed at the blind, the most common are those that rely on hearing, followed by those relying on touch, while, in the last couple of years, haptic devices have also been gaining popularity [19].

In this paper, several realizations of computer educational applications that use speech technology for desired interaction with visually impaired pupils are presented. These applications can thus be classified as audio games. During their adaptation, the focus has been on the creation and processing of information in audio form in order to produce right and timely interaction. It is a serious challenge to generate or adapt games to the needs of the disabled [20, 21] since the experience acquired by the player during gameplay varies from game to game and from person to person [22]. The research concerning educational applications based on speech technologies for Serbian represents the initial results in automatic game adaptation to user's needs in line with techniques and methods used in artificial intelligence domain.

The paper is divided into three main sections.  Section 2 outlines the information on speech technologies developed for the Serbian language. The following section covers the presentation of educational applications based on speech technologies along with the accomplished adaptations. The final two sections provide a discussion about achieved results and an overview of the plans for further research.

## 2. Speech Technologies for Serbian

Speech technologies have achieved impressive quality for some languages over the past few decades. Algorithms for both automatic speech recognition (ASR) and text-to-speech (TTS) are constantly being developed and refined, and new approaches are being defined in order to make speech technologies adaptable to different devices and acoustical environments [23, 24]. The algorithmic part of developing speech technologies is universal, while the resources are, naturally, language dependent and need to be acquired for each language. Collecting speech and language resources is a very time-consuming task, which requires domain knowledge. These are the main reasons why many languages are still underresourced in this context.

Speech technologies for Serbian have been developed over the past decade and a half at the Faculty of Technical Sciences of the University of Novi Sad, in cooperation with the company “AlfaNum” from Novi Sad. During this period, a respectable amount of data for training ASR and TTS systems has been acquired [25], and both technologies are still being constantly improved by introducing new techniques and gathering new resources. The quality of ASR and TTS that is sufficient for most practical applications has been reached several years ago and, even though further research and development are needed in order to follow the state of the art, the research is now mainly focused on creating natural-like dialogue systems. The rest of this section gives a brief overview of the ASR and TTS technologies for Serbian.

### 2.1. Automatic Speech Recognition for Serbian

The problem addressed by an ASR system is converting speech as an audio signal into a sequence of words in textual form. The main segments of an ASR system are acoustic models, a pronunciation model, and a language model [26]. Acoustic models encapsulate acoustic representations of phonemes; pronunciation model (pronunciation dictionary) represents the relations between words and the corresponding phonemes, while language model holds the information about syntactic rules.

The acoustic models, which are used within ASR for Serbian, are based on hidden Markov models (HMMs) and Gaussian mixture models (GMMs). Each triphone is represented by several HMM states, from two for R and schwa up to six for stressed vowels. Tree based clustering procedure [27] is used in order to find similar states which are then represented by a unique set of parameters, which, in turn, reduces the computational complexity of the models significantly. The Gaussians are modeled by using full covariance matrices, which lead to more accurate acoustic representations [28] at the cost of increasing the computational complexity of log likelihood calculation. The number of Gaussians per state is determined dynamically by cross-validation and ranges between two and ten. This problem can be overcome to an extent by using either of the techniques presented in [29] or [30]. The feature vector, which defines the acoustic models of triphones, consists of 15 mel-frequency cepstral coefficients (MFCCs), normalized energy, and their derivatives. Feature vectors are extracted every 10 ms from 30 ms speech segments centered around extraction time instants [25].

The pronunciation model for ASR relies on the pronunciation dictionary created for TTS [25]. Mapping words to phonemes appears to be relatively easy for Serbian because of its orthography, which is very close to phonetic, but since it is necessary to distinguish between stressed and unstressed variants of vowels, the pronunciation dictionary had to include accent information as well.

When it comes to language modeling, inflective languages such as Serbian require vast training corpora to be collected in order to obtain quality language models since the morphology of these languages is very complex. Currently, training corpora for Serbian contains around 20 million tokens altogether, which is insufficient for most applications. Therefore, a hybrid language model was created in order to overcome the problem of insufficient training data [31]. This hybrid model combines a word-based, lemma-based, and morphologic class-based model in a log-linear manner.

The recognition process is based on the token-passing algorithm [25]. The most important parameters for decoding, which define the search space, are the number of words that are expected to be recognized (the recognition vocabulary), the number of their pronunciation variants, and the number of HMM states in the acoustic models. Naturally, pruning techniques are applied in real-time recognition in order to reduce the search space by discarding the least probable hypotheses.

### 2.2. Text-to-Speech Synthesis for Serbian

There are currently two mainstream approaches to building a TTS system. One of them is concatenative approach [32], and the other one is parametric, based on hidden Markov models [33, 34]. Both have been implemented for Serbian, but currently the concatenative concept offers a higher quality of synthesized speech, which was the reason for choosing the concatenative TTS for adaptation of educational applications to the blind pupils. The speech synthesis process can be divided into high-level and low-level synthesis.

High-level syntheses within AlfaNum TTS, including phonetization, part-of-speech (POS) tagging, and the detection of prosodic events, such as prosodic phrase boundaries and prominence from text, are carried out by expert systems. Namely, POS tagging [25] relies on a morphological dictionary containing over 4 million lexical forms, which correspond to around 100,000 lemmas. Lexical disambiguation is based on a combination of hand-written grammar rules and transformation rules automatically inferred from a corpus of 200,000 words of previously POS-tagged text. As to the detection of prosodic phrase boundaries and prosodic prominence from text, the current version of the system relies exclusively on hand-written rules. Since the problem of predicting focus or deaccentuation from POS-tagged text is very complex, the system is restricted to assigning prominence markers only in some of the most obvious cases.

## 3. Educational Applications for Blind and Partially Sighted Pupils

In order to increase the quality of life of the blind and partially sighted persons, special attention needs to be paid to their education at a very early age. In early childhood, it is easier for them to accept new methods of learning and dealing with different problems. One of the most important requirements for the successful introduction of new challenges to children in general is to keep them interested in the subject. Since all children have different skills and interests, this is usually a very hard task for the people involved in their education. It is obvious that the best way to keep pupils interested in a particular subject matter is by presenting it in the context of a game. This is especially important for some particular subjects such as geometry, which is generally unpopular among the pupils and particularly difficult to comprehend for pupils with visual impairments. Furthermore, memory exercises are of great importance in the early education, but they represent a challenge when it comes to keeping the pupils interested.

The main problem that has to be addressed when developing an educational game is adapting the difficulty of tasks to individual skills of pupils. This is usually done by implementing several levels of difficulty and leaving it to the pupils or their teachers to choose an appropriate level. An unsupervised version of this would be to decrease or increase the difficulty of a task automatically, based on the previous results of each pupil.

Another important issue related to educational games and education in general is allowing the pupils to express their creativity. Most computer applications offer tasks with somewhat deterministic solution paths. Allowing the children to create their own tasks encourages them to be creative and increases their interest in the task itself.

Adapting educational games for children with visual impairment is by no means a trivial task. In order to understand the problem, a number of development iterations and tests need to be conducted. The feedback information from the pupils is essential for the development of successful applications. The introduction of speech technologies created a completely new research area related to the development of aids for blind persons. Development of educational aids based on speech technologies for blind children is a relatively young research field, and the research process in this field is relatively slow because of the need for constant testing of the applications, which is usually not easy to organize.

Having in mind the abovementioned problems and techniques used to overcome them, first educational aids for visually impaired pupils based on speech technologies for Serbian have been developed. They will be described in detail in the rest of this section.

### 3.1. *Lugram*

**Figure 1:**
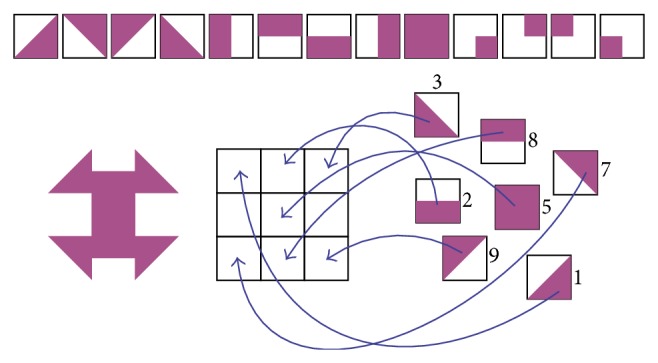
Basic constituent elements and an example of a* Lugram* task.

*Lugram* is a geometrical puzzle game. The main goal of the game is to construct a given geometric figure using the offered constituent elements. A* Lugram task* appears as a geometric figure created in a square matrix of size 3 × 3, 5 × 5, or 7 × 7 square-shaped elements. The basic version, in which* Lugram* tasks are presented in a matrix of 3 × 3 squares, has been applied in working with pupils of a regular primary school (grades I–IV). In the basic setting, they contain square, rectangle, or triangle shapes, as shown in Figure 1.

#### 3.1.1. The Basic Software Version (for Sighted Persons)

**Figure 2:**
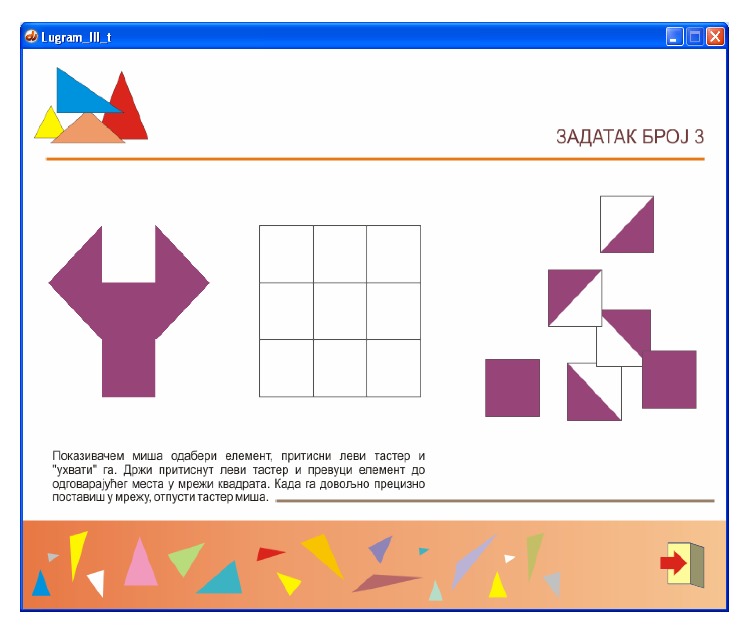
The interface layout of basic version of the game module.

The basic software version of* Lugram* operates through three modules: a* module for creating tasks*, a* module for creating constituent elements*, and* a game module*. The game module presents all constituent elements needed to form the target figure. The constituent elements “float” in the selection area, ready to be dragged and dropped into a grid where the target figure is to be formed (Figure 2). The tasks are deployed into three levels of complexity. Increasing the game complexity increases the number of different elements that form the target figure.

**Figure 3:**
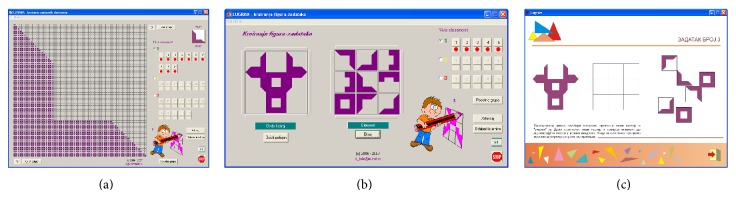
A* Lugram* task after one modification of standard constituent elements.

*Module for creating constituent elements* (Figure 3(a)) and* module for creating tasks* (Figure 3(b)) allow the creation of various tasks. The game module and the module for creating tasks receive the information about the constituent elements from the output files of module for creating constituent elements. The game module (Figure 3(c)) reads the positions of the elements in the task matrix from the output file of the module for creating tasks.

In the initial phase of implementation aimed at pupils attending a regular primary school, pupils use only the game module of* Lugram*. After gaining experience and skills in the game, they start to use the module for creating tasks, which is equipped with the initial set of constituent elements. In the last phase, pupils also use the module for creating constitutive elements. Thus, they become active participants in the process of creating new* Lugram* tasks.

**Figure 4:**
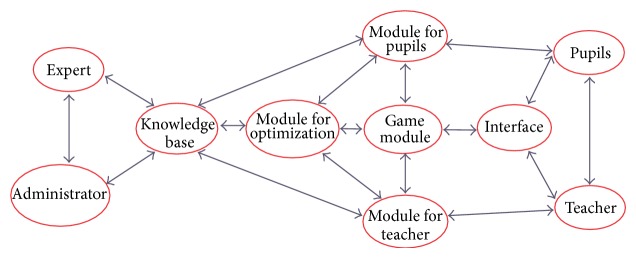
Concept idea of* Lugram* intelligent tutoring system.

**Figure 5:**
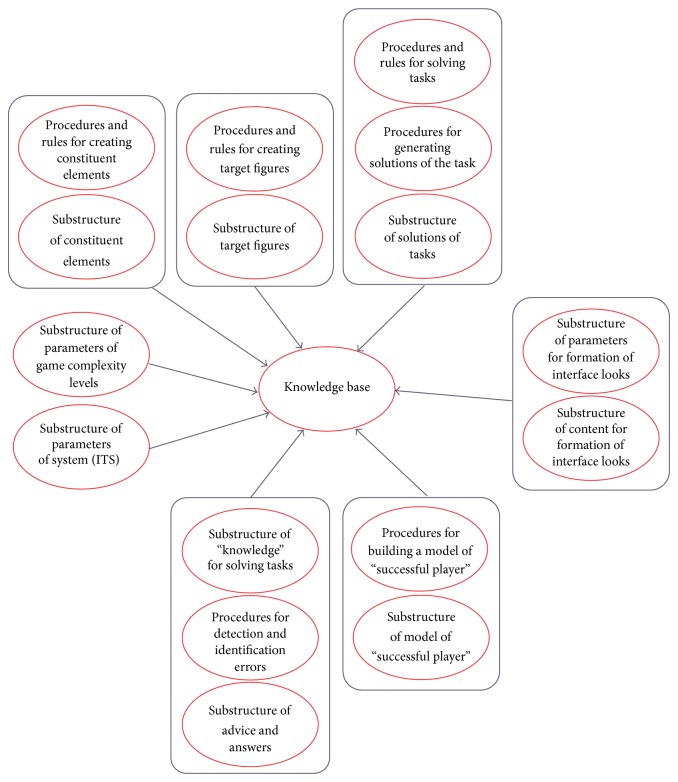
Concept of knowledge base for* Lugram.*

The described modules represent the initial step towards the realization of the idea to form an Intelligent Tutoring System (ITS) for* Lugram* (Figure 4), by involving users to improve that process. The collection of* Lugram* tasks represents a small part of the future knowledge base of* Lugram-ITS* (Figure 5).* Lugram-ITS* should operate on the principle of coaching, that is, following the flow of the game and including educational content (error messages, advices, templates, examples, etc.) in moments when it is needed and thus leading pupils to success in solving the tasks.

The proposal is based on the principles of developing intelligent tutoring systems based on semantic networks. It is intended as a preliminary blueprint for ITS prototype [35], whose implementation would form a model of a “successful* Lugram* player” who will teach other players and learn from them.

Demanding in terms of methods and forms of work and teaching tools and materials, teaching geometry becomes even more demanding when working with blind pupils. Dominantly visually designed modalities need to be replaced with modalities accessible to that population.* Lugram* is suitable for adaptation to the blind children. The aim of the adaptation is to place the basis in further work for achieving results similar to the previously described.

Keeping in mind the concept of the future* Lugram-ITS*, the development of modules adapted for blind children is supposed to contribute to the improvement of its structural and procedural segments. The application of speech technologies for Serbian language in the development of the adapted module opens new possibilities in terms of control and management when talking about the whole* Lugram-ITS*. The following section provides a brief overview of the experience and benefits acquired during the adaptation of* Lugram* for the purposes of working with blind children.

#### 3.1.2. The Basic Version Adapted for Blind Pupils

For solving* Lugram* tasks, the ability to create a mental representation of a complex geometric figure is necessary. If the sense of sight is missing, the dominant support for resolving this problem is lost. The game module is transformed into an audio game module, replacing the visual predominance by auditive. The elements of the game are represented by voice messages and sound effects. The files with voice messages and instructions were prepared by using the text-to-speech (TTS) technology for the Serbian language [3, 36, 37]. The game can be played by using only a standard PC keyboard (as a tactile device) and by voice commands (using ASR server application) [38].

**Figure 6:**
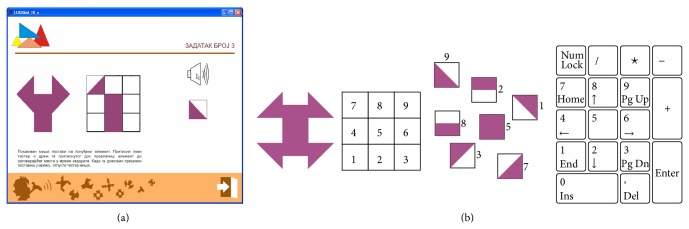
(a) Constituent elements offered one at a time. (b) Numeric keypad and redefined task matrix.

The visibility of screen content is provided nevertheless, in order to allow teachers to simultaneously assist multiple blind pupils. Constituent elements of the puzzle are static in space and offered in random order, one at a time from a set of corresponding constituent elements (Figure 6(a)), instead of being presented all at once [39].

Positions of matrix elements are aligned with positions of the numeric keys of the numeric PC keypad (Figure 6(b)). For executing moves and navigation through the game, the player uses the keys of the numeric keypad (number, Enter, and plus) as well as some other keys (ESC and spacebar). Selection of the game level and tasks, as well as positioning the constituent elements in the grid for solving a task, is done by pressing the appropriate key of the numeric keypad.

**Figure 7:**
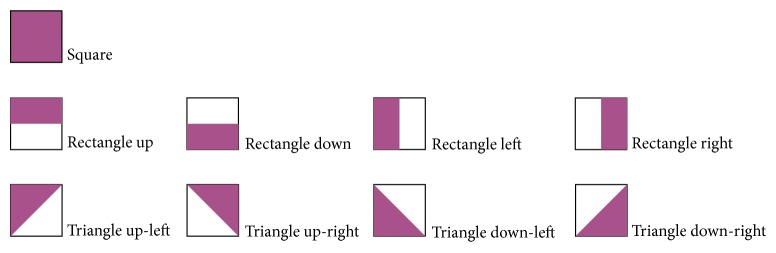
Description of the constituent elements by voice messages.

Voice instructions are separated by the sound effect, separator, in order to highlight the guidelines in user's participation in the game. Voice instructions are reduced after the first successfully solved task. The number of wrong moves in solving a task is limited to 9. Each press on the keypad buttons is accompanied by an appropriate sound effect. Constituent elements, presented descriptively by voice messages, contain up to three words (Figure 7).

*Lugram* prototypes for blind users were first tested by 10-year-old sighted pupils. They were allowed to play* Lugram* guided only by voice instructions, without seeing the display monitor and using only a keyboard. After working, pupils were asked to describe (using the introduced standard terms) the target figure which the teacher drew on the blackboard, and pupils successfully completed this activity. After the described tests, tests with two blind children of different ages and intellectual and motor skills were organized. The first child was a blind 12-year-old pupil, whereas the younger pupil was 8 years old. The older pupil was able to use a computer unaided, with the help of screen readers and speech synthesis, had previous knowledge of geometry, and was able to test the* Lugram* prototype for the blind with success. The younger pupil was of slightly weaker motor skills and had a limited knowledge of geometry, and, for these reasons, the help with the tactile model of* Lugram* was of particular importance to him. He solved only very simple tasks at the first level of complexity. After the initial tests with two blind pupils, testing in a specialized primary school for the blind children was organized. Testing was organized with six 10-year-old pupils, who did not have any other major disability and had prior knowledge of geometry required to play* Lugram*. The tests showed that the pupils were not quite familiar with terms left and right and up and down and had difficulty in understanding the position/orientation of the constituent elements [39, 40].

In general, the experience gained while testing the described prototypes led to the following conclusions: (a) the number of different constituent elements in the tasks should not be higher than three (full square, rectangle, and triangle); (b) for tasks of the first level of difficulty, only the element “square” should be used; (c) the second level may include orientation within the constituent elements (left-right, up-down), introducing the rectangle; (d) the third level of the game tasks involves orientation (up-left, up-right, down-left, and down-right) at the level of constituent elements, introducing the triangle.

**Figure 8:**
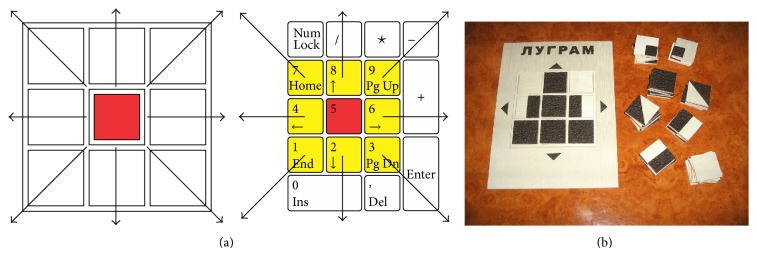
(a) Correlation: tactile model, numeric keypad. (b) Wooden tactile model.

The final version of the* Lugram* tactile model is made of wood and embossed rubber (Figure 8(b)). This has ensured that the concept of the game, the target figure matrix, and the appearance of the constituent elements can be introduced to the pupils more easily. The tactile model also plays a very important role in training the pupils to use a standard PC keyboard as a tactile device (Figure 8(a)).

**Figure 9:**
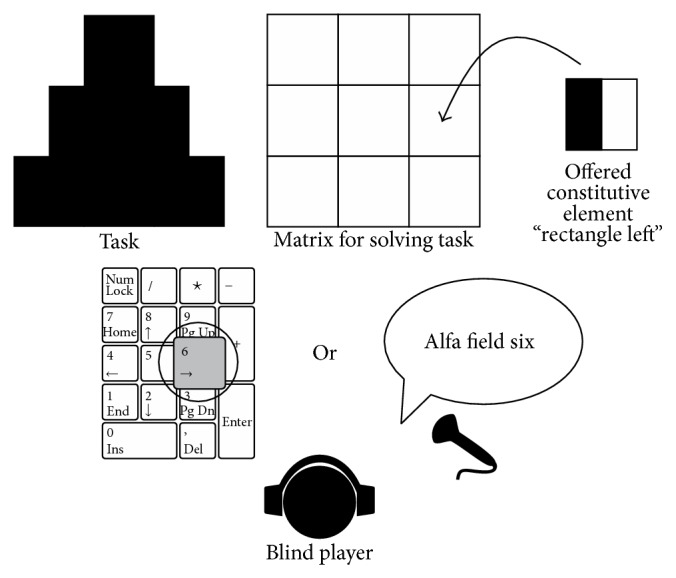
The move of the blind player.

In the first contact with* Lugram*, blind pupils follow the verbal task presentation, by tactile “reading” of the task. The program describes the content of the figure (task) matrix by describing the content and position of each of the nine constitutive elements. The user is supposed to remember that information, for example, “The task number 4,” “Target figure,” “Field 1: square, Field 2: square, Field 3: square, Field 4: rectangle right, Field 5: square, Field 6: rectangle left, Field 7: empty field, Field 8: square, Field 9: empty field.” The compositional elements are offered one at the time, using the same descriptions as in the setting of the task, for example, “The inventory of the constituent elements,” “Element: rectangle left.” In case of the abovementioned examples of the task, the user should play the move by pressing the keypad button with number 6 (Figure 9). In describing the task matrix, the program refers to empty constituent elements as “empty fields.” Thus, the “empty field” represents the information about the layout of the target figure, but later it will not be presented in the inventory as a constituent element, as opposed to the basic version of the program.

While using the game module, the user can use voice commands in the Serbian language. The game module is connected to the ASR server application for Serbian [38]. The grammar of voice commands for* Lugram* implies a required keyword (in this case, it is the word “*Alfa*”). Voice commands are short and simple: “Alfa level one,” “Alfa task three,” “Alfa field five,” “Alfa end program” ….

In the previously described example of a* Lugram* task, the user plays the correct move by using the speech command: “Alfa field six” (Figure 9). The content of voice instructions is focused on a new way of navigating through the game. Other characteristics of the voice elements of the application remain the same as in the module with navigation realized by using the keyboard.

Regardless of the way of navigation through the game, the program handles the moves in the same way. The content of feedback differs only in the part that refers to the manner of conducting the following activities in the game (by keyboard or voice command).

**Figure 10:**
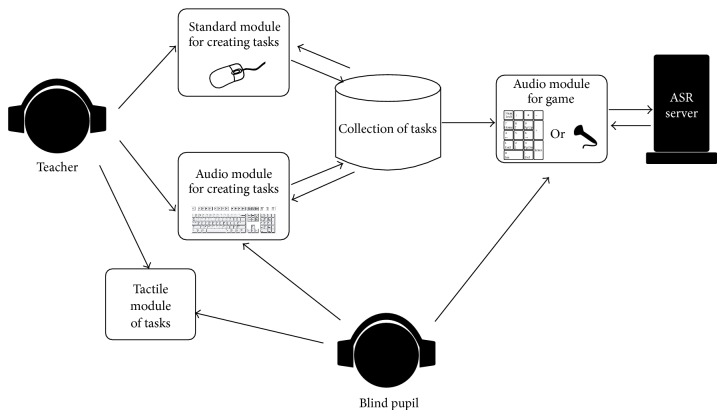
Multimodal approach.

By gaining experience through the multimodal approach (Figure 10) of solving the initial groups of tasks, the pupils get prepared to use the version of the game in which the only tactile element is the PC keyboard.

**Figure 11:**
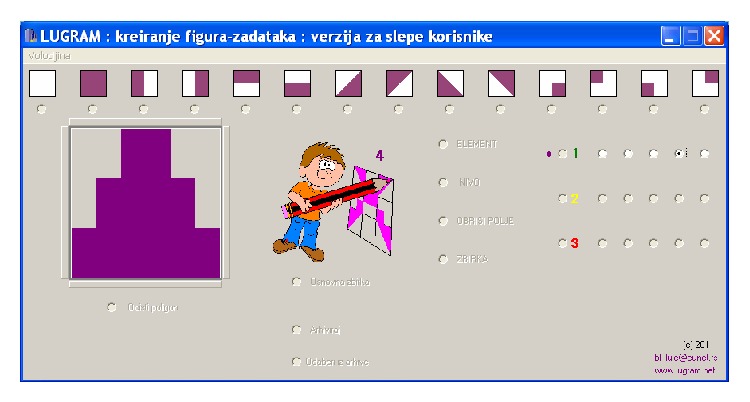
The interface of the audio module for creating* Lugram* tasks.

For creating tasks, either the standard module or the module adapted for the blind can be used. Preparation of tasks by using the standard module can be performed only by sighted persons, but attention needs to be paid to harmonizing the tasks with the concept designed for working with blind children. At the moment, working with the adapted audio module for creating tasks (Figure 11) is possible only by using the keyboard as a tactile device. This module involves a larger number of keys than the game module. The audio module for creating tasks uses a part of voice content inherited from the game module, and a considerable quantity of new content as well, for example, “Key F5 has been pressed. Recording of the task number 5 is running on the hard drive of your computer” and “The level 3 has been selected. Press Enter if you want to view tasks” [41].

The population of those who can prepare* Lugram* tasks is thus extended to the blind. This means that blind pupils who were successful as game players get the opportunity to create tasks, like their peers with regular sight.

### 3.2. *anMasterMind*

Application* anMasterMind* represents another useful instrument in the research related to the education of the visually impaired. It is based on a well-known code-breaking game called Master Mind. In the pen-and-paper version of this game, the player who creates a task (the* codemaker*) composes an ordered set of four symbols by choosing from a set of six symbols. The symbols chosen to compose a task may not be mutually different. The object of the game for the player who solves the task (the* codebreaker*) is to find out the combination of symbols composed by the codemaker. To that purpose, the codebreaker iteratively names combinations of symbols and uses the feedback information from the codemaker in order to get closer to the solution. The codebreaker is given six (or, in some versions, seven) tries to solve a task. The feedback from the codemaker includes information on how many symbols contained in the codebreaker's proposition are also contained in the solution, as well as how many of those symbols are in the right positions. The game is intended to serve as a memory exercise for the blind.

In the computer version of this game, codemaker is the computer. The computer game* anMasterMind* was created for both the visually impaired and persons with regular sight. Therefore, a graphic user interface (GUI) has been implemented, and it is shown on the screen even in the mode for the blind. This was convenient for the research including blind pupils, because the instructor could follow the flow of the game.

**Figure 12:**
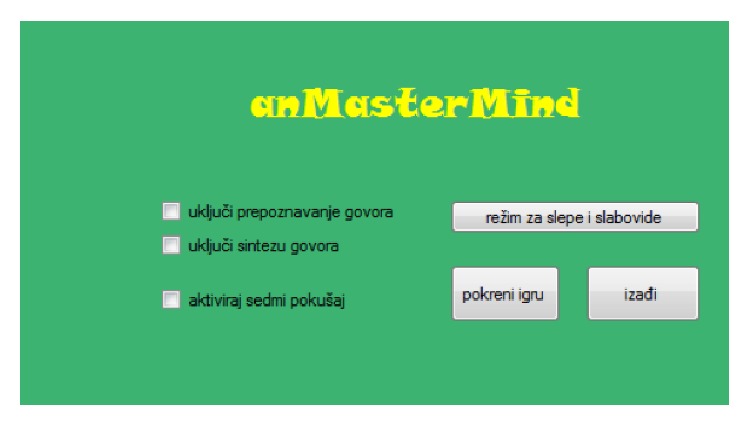
The initial window of the* anMasterMind* application.

The initial window, displayed upon starting the application, is shown in Figure 12. It contains buttons for starting and exiting the game. The checkboxes serve to activate ASR and/or TTS and to activate the seventh try (the default number of tries is six). The mode for the visually impaired can be activated by pressing the appropriate button or by using the keyboard shortcut “space,” which is more convenient for the blind persons. If the mode for the visually impaired is activated, the configuration of the checkboxes is ignored and both ASR and TTS are activated, as well as the seventh try. Furthermore, special voice commands and voice messages, which are not available in the regular mode when ASR is activated, are enabled. In the rest of this section, the description of the application will refer to the mode for the visually impaired.

**Figure 13:**
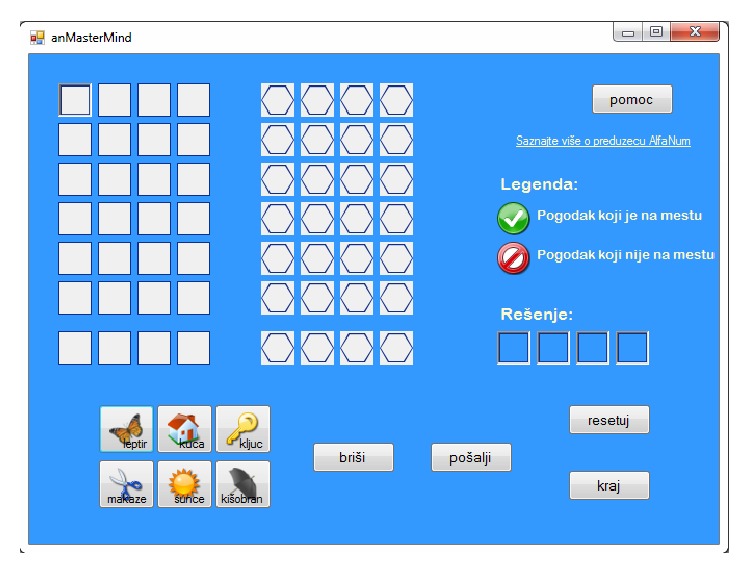
The main window of the* anMasterMind* application.

After the configuration has been set, the main window is displayed, as shown in Figure 13.

The main window contains buttons for resetting and exiting the application, as well as the help button. There is also a button used for erasing a previously chosen symbol, as well as a button for requesting feedback when the player completes a combination of symbols. Furthermore, there are six buttons corresponding to the set of available symbols: butterfly (*leptir*), house (*kuća*), key (*ključ*), scissors (*makaze*), the Sun (*sunce*), and umbrella (*kišobran*). The symbols chosen by the player are displayed in the corresponding fields within the table on the left side of the main window. Next to this table, there is a table in which the feedback information is displayed in the form of combinations of two symbols (not counting the empty fields which symbolize misses), one that marks a hit out of place, and another which marks a hit in place.

Each of the buttons within the main window can be activated by a mouse click, which is an option useful for the partially sighted pupils, while blind pupils can use voice commands instead. Furthermore, there are voice commands that do not have corresponding visual representations on the main window. These commands are implemented in order to allow the player to obtain information about the previous tries and feedback within a game round.

**Figure 14:**
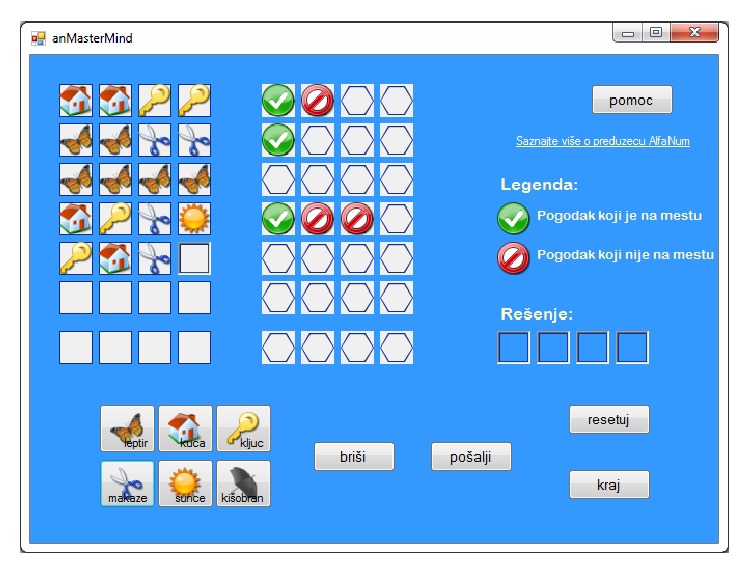
The* anMasterMind* main window, game in progress.

Among these are commands such as “read the previous try,” commands for retrieving information on any previous try by number, for example, “read fifth try,” the command for reading the entire game round history, “read all,” and the command for reading the current selection of symbols (even if not complete), “read.” For each of the commands, a set of several most likely strings of words, which may be used, is supported. This is accomplished by defining an appropriate grammar for the speech recognizer, and the grammar for this application was defined as follows (a rough translation into English is given, disregarding the existence of the inflected forms of nouns and gender-dependent forms of numbers, which exist in the original Serbian version):

 command1 = THE END ∣ EXIT ∣ CLOSE ∣ (NEW GAME) ∣ RESET ∣ HELP;
 command2 = BUTTERFLY ∣ HOUSE ∣ KEY ∣ SCISSORS ∣ SUN ∣ UMBRELLA ∣ SEND ∣ SUBMIT ∣ ERASE ∣ DELETE ∣ BACK;
 command3a = TWO ∣ THREE ∣ FOUR;
 command3b = BUTTERFLIES ∣ HOUSES ∣ KEYS ∣ PAIRS ∣ SCISSORS ∣ SUNS ∣ UMBRELLAS;
  
 command4 = READ;
 command5 = FIRST ∣ SECOND ∣ THIRD ∣ FOURTH ∣ FIFTH ∣ SIXTH ∣ SEVENTH ∣ (GAME FLOW) ∣ (HISTORY) ∣ ALL ∣ EVERYTHING ∣ (FROM THE BEGINNING);
 command6 = TRY ∣ ATTEMPT;
  
 rule1 = $command1;
 rule2 = {($command3a $command3b) ∣ $command2};
 rule3 = $command4 [$command5 [$command6]];
  
 do = $rule1 ∣ $rule2 ∣ $rule3;
 main = [$do];

This ensures that the communication between the user and the machine resembles natural human interaction. The feedback information in the form of synthesized speech is also composed along the lines of a natural human conversation. For example, if the user asks about the fourth try within a game round displayed in Figure 14, the feedback would be in the following form: “In the fourth try, you have selected the combination: house, key, scissors, the Sun. You have three hits, one of which is in the right position.” If the codebreaker solves the task, he/she receives a voice message, after which a new round can be started. If the codebreaker is not successful, after the seventh try a voice message informs him/her on the correct combination of symbols, which is also displayed in the four fields on the right side of the main window (this is convenient for partially sighted).

**Figure 15:**
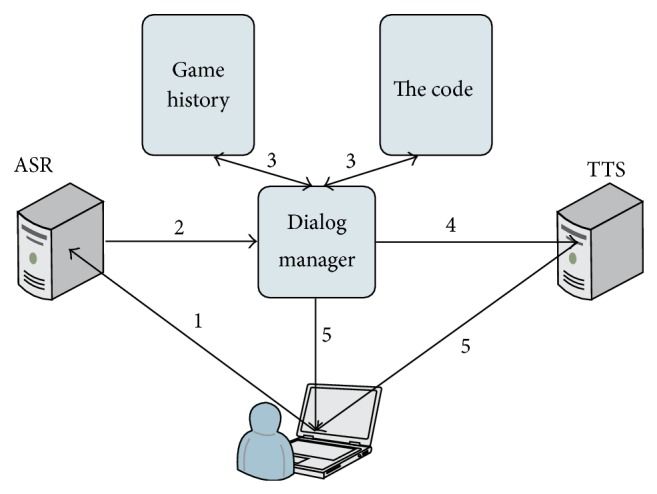
Human-machine interaction based on speech technologies: (1) a speech message is forwarded to ASR server; (2) speech recognition is applied and textual information is sent to the dialog manager (DM); (3) DM deduces the intended command and, taking into account the code and game history, executes the command; (4) in case the command was a question, the DM creates a textual message for the user and forwards it to the TTS server; (5) feedback information is presented to the user in graphic and/or speech form.

The human-machine interaction through voice messages is enabled by communication of the core application with ASR and TTS servers, shown in Figure 15 (in a simplified form).

The ASR and TTS server applications may be installed locally or on a different computer.

## 4. Initial Results and Discussion

**Figure 16:**
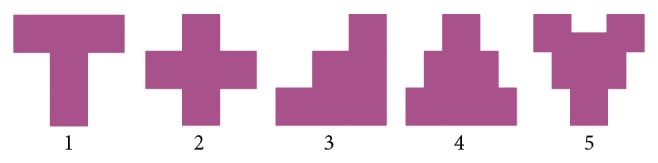
Simple* Lugram* tasks for first tests with blind pupils.

The computer game* Lugram* has been tested as a learning aid for children without visual impairment for several years and proved to be an effective educational method in that context. The impact of* Lugram* on pupils' achievements in learning geometry was analyzed. The research on a sample of 89 third-grade pupils of a regular primary school was organized. The final knowledge test has shown an increase of the success (*df* = 87, *t* = 2.15, and *α* = 0.05) in favor of the experimental group. The experimental group was using* Lugram* during the experimental period, while working on a curricular topic “Triangle” [35, 42]. The initial work with the blind and partially sighted pupils gave very encouraging results. Namely, in the case of* Lugram* application, the blind pupils were able to complete the tasks (Figure 16) after initial mentoring by their teacher.  Table 1 provides more detailed results.

**Figure 17:**
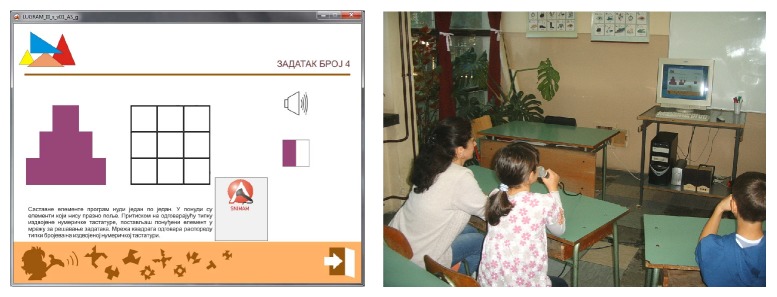
* Lugram* with added ASR support and the initial test of accepting formed voice commands; preliminary test with sighted pupils.

This resulted in motivation to develop a set of tasks of different complexity levels in order to monitor the progress of blind pupils over time, as it was done with pupils without visual impairment. Initial tests of* Lugram* application that supported ASR, which were conducted with blind pupils, confirm the acceptability of introduced voice commands that the preliminary tests with sighted pupils showed in Figure 17 [43].

The analysis of the log files showed a high level of accuracy in terms of speech recognition when voice commands were issued by children. Benefit from such an outcome is not only the possibility of further development and management of* Lugram* application by using the voice commands in Serbian, but also the fact that they can become a part of the future knowledge base* Lugram-ITS*.

The* anMasterMind* application proved to be an interesting method for memory exercise. Five pupils of age between ten and twelve participated in the initial study. Four out of five were able to solve a given task at least once in several tries [44].  Table 2 provides details of the test results. Keeping in mind that the tasks were not equally difficult to solve, no correlation can be conclusively derived between the result and the number of times the user asked to hear the game history or just one of the previous tries. Further tests are clearly necessary, but the initial results show that* anMasterMind* can be viewed as a method for memory exercise that can be further improved by monitoring the progress of the pupils.

Clearly, there is a need to define different levels of task complexity within* anMasterMind* application, as it was done with* Lugram*. This can be done by reducing the set of used symbols, by classifying the tasks by rules based on empirical knowledge, or by extending the number of tries.

## 5. Conclusion and Further Research

Incorporation of speech technologies into both educational applications described in this paper has enabled research concerning pupils with disabilities. However, as is the case with most of the other research aimed at improving the quality of life, any quantitative evaluation has very little meaning. The process of introducing educational applications is iterative and requires constant feedback from the pupils and their mentors in order to fine-tune the complexity level of individual tasks, as well as to introduce new features to the applications in order to improve the learning experience. The success of this process can only be measured in the context of acceptance of the educational applications by the pupils and their progress in solving the tasks that require them to create mental representations of objects and memorize the order in which the objects are presented to them.

The general conclusion of this research is that the visually impaired pupils should be continuously motivated to make progress in their understanding of the world and solving tasks that are imposed upon them by everyday life, starting at early age and by introducing learning tools based on audio information. Applications based on speech technologies can be of great help in this process and it is crucial that they are age-appropriate and that different levels of complexity are defined in order to meet the needs of pupils with different degrees of disability. The initial success of introducing the ASR and TTS technologies to the visually impaired pupils resulted in motivation for further research in this field.

The research concerning improvement of the speech technologies for Serbian is the basis for creating advanced tools and applications, which would serve to help children and adults with different types of disabilities to overcome the problems that they face. Current research related to speech recognition for Serbian includes experiments using the* Kaldi* toolkit [45]. The aim of these experiments is to create an efficient large vocabulary speech recognition system, which could be used for dictation but would also enable even more natural-like dialogue between humans and machines. Ongoing research related to Serbian TTS is aimed at creating expressive text-to-speech and is still in the database preparation phase [46].

Further improvements to* Lugram* and* anMasterMind* are inspired by the information collected during initial tests, which have been mentioned within the previous section. The application of speech technologies for Serbian has enabled a broader approach to the procedural and structural part of the* Lugram-ITS* and improved the quality of its current concept, which is intended only for sighted users.

Furthermore, valuable information was collected through communication with pupils, who described the features they would like the applications to have, mostly concerning the dialog form. Moreover, many other educational games may be adapted to visually impaired pupils by incorporating speech technologies.

## Figures and Tables

**Table 1:** Results of *Lugram* tests with six blind pupils (age 10).

	Tasks														
	1			2			3			4			5		
	*t* (mm:ss)	Teacher help	err	*t*	Teacher help	err	*t*	Teacher help	err	*t*	Teacher help	err	*t*	Teacher help	err
1	2:38	Y	3	1:52	Y	2	2:56	N	4	4:24	Y	5	4:11	N	7
2	3:11	Y	4	3:10	N	3	3:35	N	4	4:27	Y	6	6:17	N	5
3	2:48	Y	3	2:43	Y	2	2:50	N	3	4:39	Y	5	4:50	Y	5
4	3:50	Y	5	3:40	Y	5	3:20	N	4	6:12	Y	7	5:48	Y	4
5	2:45	Y	2	2:33	Y	3	2:58	N	3	5:40	Y	3	4:45	N	5
6	2:28	Y	3	2:29	N	2	2:19	N	5	4:56	Y	3	4:29	N	6

**Table 2:** Results of *anMasterMind* tests with five blind pupils.

Age	Sex	Time	Task complete	Final try	Requests for history	Requests for a single try
10	M	3	Yes	6	0	0
		6	No	—	0	1
		5	Yes	6	0	2
		3	Yes	6	0	0
		10	No	—	1	1
		8	No	—	1	2
		8	Yes	6	1	1

11	F	8	No	—	2	1
		5	Yes	6	2	1
		4	No	—	2	3
		6	No	—	2	0
		9	Yes	6	2	3
		9	No	—	1	3
		9	Yes	4	2	1

10	M	5	No	—	1	2
		7	Yes	6	2	0
		5	No	—	2	0
		3	Yes	6	1	0
		8	Yes	7	3	1
		9	No	—	3	3

12	F	15	No	—	2	2
		9	No	—	1	1
		10	Yes	7	3	3
		8	No	—	2	2

12	M	13	No	—	3	1
		10	No	—	2	1
		9	No	—	2	2
